# Expression Analysis of miRNA Profiles in Colorectal Cancer with a Bioinformatics Approach: An Emphasis on miR-4295, miR-4720-5p, miR-4773, and miR-6831-5p

**DOI:** 10.3390/diagnostics16040614

**Published:** 2026-02-19

**Authors:** Recep Eskin, Turkan Gurer, Alper Aytekin, Filiz Ozbas Gerceker

**Affiliations:** 1 Department of Biology, Faculty of Art and Science, Gaziantep University, Gaziantep 27310, Turkey; recepeskin@gmail.com (R.E.); filiz.gerceker@gmail.com (F.O.G.); 2 Department of General Surgery, School of Medicine, Gaziantep University, Gaziantep 27310, Turkey; aytekinalper83@hotmail.com

**Keywords:** colorectal cancer, miR-4295, miR-4720-5p, miR-4773, miR-6831-5p, RT-qPCR, bioinformatics

## Abstract

**Background/Objectives:** This study aimed to determine the potential roles of miR-4295, miR-4720-5p, miR-4773, miR-6831-5p, and miR-7161-5p in colorectal cancer by evaluating their expression levels in matched tumor and adjacent non-tumor tissues from 86 patients. **Methods**: A total of 172 samples were analyzed, and the associations between miRNA expression levels and clinicopathological characteristics were assessed, along with correlations among the miRNAs. Functional enrichment analyses, including GO and KEGG pathway evaluations, were performed using DIANA-mirPath v.3 to characterize biological processes and signaling pathways associated with the predicted target genes. **Results:** The results showed that miR-4295 and miR-4720-5p were significantly upregulated in tumor tissues, while miR-4773 and miR-6831-5p were significantly downregulated (*p* < 0.001). No significant difference in miR-7161-5p expression was observed between tumor and non-tumor tissues (*p* = 0.877). KEGG analysis indicated that miR-4295, miR-4720-5p, miR-4773, and miR-6831-5p regulate genes involved in the TGF-β, mTOR, ErbB, FoxO, and endocytosis signaling pathways. **Conclusions**: These findings suggest that miR-4295 and miR-4720-5p may have oncogenic functions, while miR-4773 and miR-6831-5p may have tumor-suppressing functions, and that this relationship may contribute to the development of colorectal cancer.

## 1. Introduction

Colorectal cancer (CRC) represents a major malignancy of the gastrointestinal tract and ranks among the leading cancer types in terms of global incidence in both men and women. It represents the third leading cause of cancer-associated death in males and the fourth leading cause in females [1]. Globally, approximately two million new cases of CRC are diagnosed annually, while almost a million people die from this disease [2,3]. CRC shows diversity among individuals in the mechanism of formation as well as at the cellular and molecular level [4]. A number of risk factors are known to contribute to the development of colorectal tumors, including older age, family history, Western-style diet, smoking, excessive alcohol consumption, sedentary lifestyle, diabetes, obesity, and ulcerative colitis. However, the most serious risk factor is age; adolescents and elders are particularly more susceptible [5,6]. CRC is a complex and heterogeneous disease with multiple genetic mutations and epigenetic modifications. The initiation of CRC is driven by a series of interconnected genetic and epigenetic mechanisms, marked by aberrant oncogene signaling and suppression of tumor suppressor pathways within the colonic epithelium. Such alterations facilitate the transition from colorectal adenomas to malignant adenocarcinoma [7]. Epigenetic regulation is essential for maintaining normal cellular functions, and disruptions in these processes can contribute to cellular dysregulation and the development of various malignancies, including CRC [8,9,10,11]. Therefore, microRNAs (miRNAs), playing an important role in physiological and pathological processes, can cause critical changes in target genes as regulators of nearly one third of human genes [12].

miRNAs are non-coding endogenous negative regulators of gene expression, consisting of approximately 20–24 nucleotides and representing a class of evolutionarily significantly conserved molecules. The regulatory effects of these small molecules can be explained by their ability to bind with complementary mRNAs via base-pairing interactions. Two mechanisms are responsible for the functionality of these molecules. The first mechanism involves the process of mRNA degradation, while the second one operates through the inhibition of mRNA translation. miRNAs serve as key regulators of numerous biological processes, contributing to the maintenance of cellular homeostasis through their involvement in cell growth, development, metabolic regulation, programmed cell death, immune responses, intercellular signaling, and differentiation [13]. The results of numerous studies have highlighted the detection of different levels of miRNAs in various cancers, including CRC. These molecules have been implicated as oncogenes or tumor suppressors in the process of carcinogenesis through several mechanisms [14,15,16]. More than 50% of miRNA genes are located on the cancer-associated genome and regulate many important signaling pathways related to CRC pathogenesis [17]. miRNAs affect the expression of several genes involved in key pathways, such as transforming growth factor beta (TGF-beta), mechanistic (mammalian) target of rapamycin (mTOR), ErbB receptor (ErbB), Janus kinase/signal transducer and activator of transcription (JAK/STAT), vascular endothelial growth factor/vascular endothelial growth factor receptor (VEGF/VEGFR), epidermal growth factor receptor/mitogen-activated protein kinase (EGFR/MAPK), and hippo signaling pathway with yes-associated protein (Hippo-YAP), that contribute to CRC formation [18,19,20]. In numerous experimental studies, miRNAs appear to have a significant role in cancer formation, prognosis, and progression. Therefore, they attract attention as agents that can be used as therapeutic targets [21,22].

Many studies have reported significant differences in miRNA expression levels between normal and tumor tissues in various cancer types [23,24]. In a study investigating the link between CRC and miRNAs, Gurer et al. (2022) reported a significant downregulation of miR-485-3p and miR-4728-5p in tumor tissues [25]. In a recent study, downregulation of miR-143, miR-145, miR-574-3p, and miR-133b, considered to have a potential tumor suppressor role in CRC, was observed, and these miRNAs were defined as valuable biomarkers for diagnostics and prediction [26]. In another study, Susanti et al. reported an increased expression of miR-135a/b-5p and miR-494-3p, involved in adenomatous polyposis coli (*APC*) gene silencing, which might have potential use in early diagnosis of CRC as well as transcriptomic anti-mir-based therapy [27]. In a further study, the upregulation of miR-135b and miR-182 in CRC tissue lines was shown to promote proliferation via the phosphoinositide 3-kinase/protein kinase B (AKT) (PI3K/AKT) pathway [28]. There are numerous studies showing dysregulation of different miRNAs in various types of cancers. miR-4295 expression was examined in osteosarcoma [29], glioma [30], thyroid [31], gastric [32], kidney [33], and pancreatic cancers [34]; miR-4720-5p in hepatocellular carcinoma [35] and intramucosal gastric cancers [36]; miR-4773 in gastrointestinal stromal tumor [37] and breast cancer [38]; miR-6831-5p in bladder [39], pancreatic [40,41], breast [42], prostate [43], and gastric cancers [36].

The selection of miR-4295, miR-4720-5p, miR-4773, miR-6831-5p, and miR-7161-5p in this study is based on the previous reports indicating their dysregulation in various malignancies, and the lack of studies investigating comprehensive expression data in CRC. Although these miRNAs play a role in different cancer-related signaling pathways, their potential roles in CRC have largely been uninvestigated. Therefore, this research aimed to explore the expression profiles of these miRNAs in CRC tissues and to elucidate their possible roles in colorectal carcinogenesis.

## 2. Materials and Methods

### 2.1. Patients and Tissue Samples

The present study’s sampling comprised 86 patients diagnosed with CRC who underwent the relevant surgical operations between 2018 and 2022. Prospectively matched tumor and non-tumor healthy tissues were collected from each patient, making a total of 172 samples. The adjacent non-tumor healthy tissues were collected from samples taken at a sufficient distance from the tumor margin during the same surgical resection procedure and represented histologically normal colon and rectal tissues. Importantly, no additional invasive procedures were performed to obtain control samples, and all tissue collection procedures were performed within the scope of standard surgical treatment. All surgical operations and tissue sample collections were carried out at Gaziantep University Faculty of Medicine, Department of General Surgery. The patients to be included in the study were chosen according to the fact that they were diagnosed with colon and/or rectum adenocarcinoma as a result of histopathological diagnosis and did not receive any previous chemotherapy or radiotherapy treatment. Furthermore, patients were excluded from our research according to the following criteria: those diagnosed with other types of cancer, autoimmune or inflammatory diseases and cardiovascular disease within the previous six months, active infection within the body, long-term bedridden patients, and patients unable to tolerate surgery. Within the present study, written and signed informed consent was obtained from all patients. Ethical approval for this study was obtained from the Medical Ethics Committee of Gaziantep University (approval no. 2021/148, approved date: 14 July 2021). All procedures were conducted in compliance with the ethical standards outlined in the Declaration of Helsinki. All tissue samples collected for the analysis were stored in a solution of RNAlater (Invitrogen; Thermo Fisher Scientific, Inc., Carlsbad, CA, USA) at a temperature of −80 °C.

### 2.2. RNA Isolation, Quantitation, and Evaluation

The total RNA was extracted using the mirVana^TM^ miRNA Isolation Kit (Invitrogen, Thermo Fisher Scientific, Waltham, MA, USA, AM1560) in accordance with the manufacturer’s instructions. The quantification (concentration and purity) of the isolated RNA was determined using a NanoDrop spectrometer (Maestrogen, Hsinchu, Taiwan). The RNA concentration of each sample was calculated by the ratios A260/A280 and A260/A230. Samples with an OD A260/A280 ratio between 1.8 and 2.1 were considered as highly pure RNA. Following isolation, RNA samples were preserved at −80 °C until further analysis.

### 2.3. cDNA Synthesis

Total extracted RNA was converted to cDNA using a TaqMan^TM^ Advanced miRNA Reverse Transcription Kit (Applied Biosystems, Thermo Fisher Scientific, Waltham, MA, USA, A28007). cDNAs were stored at −20 °C under appropriate conditions for use in Quantitative Real-time Polymerase Chain Reaction (RT-qPCR).

### 2.4. Amplification of miRNAs by RT-qPCR

Quantitative Real-time PCR (RT-qPCR) was performed using TaqMan^®^ Universal PCR Master Mix (Applied Biosystems, Thermo Fisher Scientific, Waltham, MA, USA, 4444557), and TaqMan^®^ MicroRNA Analyzers (Applied Biosystems, USA). miRNA-specific primers were used to calculate the relative expression levels of miR-4295, miR-4720-5p, miR-4773, miR-6831-5p, and miR-7161-5p according to the 2^−∆∆Ct^ method [44]. *RNU6* was selected as a housekeeping gene as previously reported in CRC miRNA studies [16]. Samples were run in three replicates to determine the mean cycle threshold (Ct) value for the amplification of each sample. The sequences of the primers used for each miRNA are listed in Table 1.

### 2.5. Functional Enrichment Analysis of miRNAs

DIANA-mirPath is an online tool for analyzing miRNA pathways that can support complex processes and provide precise statistics. MirPath uses experimentally validated interactions between miRNAs obtained from DIANA-TarBase or predicted targets of miRNAs (in CDS or 3′-UTR regions) provided by the DIANA-microT-CDS algorithm. It then uses advanced assembly and meta-analysis procedures to identify these interactions (predicted and/or validated). To further elucidate the biological functions of target genes affected by dysregulated miRNAs, Gene Ontology (GO) and Kyoto Encyclopedia of Genes and Genomes (KEGG) pathway enrichment analysis is performed using the DIANA-miRPath v3.0 online tool [45]. KEGG analysis is performed to predict the metabolic pathways of target genes of dysregulated miRNAs in tissue from CRC patients. A Venn diagram is generated by overlaying the target genes of miRNAs using the Venny 2.1.0 web application [46].

### 2.6. Statistical Analysis

The data processing was carried out using SPSS Software, version 22.0, developed by IBM Corp. All data were expressed as the mean ± standard deviation. RT-qPCR data analysis was conducted using the 2^−ΔΔCt^ method [46]. Initially, the Ct values for each sample were determined. Subsequently, utilizing the ΔCt (ΔCt = Ct target − Ct *RNU6*) values from tumor and non-tumor tissues, the ΔΔCt (ΔΔCt = ΔCt tumor − ΔCt non-tumor) values were calculated. Finally, the fold-change values were calculated using the 2^−ΔΔCt^ method. The statistical analysis was conducted using the Kolmogorov–Smirnov test. Given the normality of the data distribution, the ΔCt values of the following miRNAs (miR-4295, miR-4720-5p, miR-4773, miR-6831-5p, and miR-7161-5p) were compared in the tumor and adjacent non-tumor tissues using the paired *t*-test. Furthermore, the relationship between the expression levels of these miRNAs and the clinicopathological features of the patients was determined by performing Pearson chi-square (χ2) and Fisher’s exact tests. For these analyses, miRNA expression levels were grouped as high or low based on fold change values (>1 or <1), a commonly used approach in relative expression studies that utilizes the 2^−ΔΔCt^ method to distinguish between upregulated and downregulated expression patterns, as previously described in the literature [16]. This categorization was applied to facilitate exploratory subgroup analyses. Given the exploratory and hypothesis-generating nature of this study, no formal adjustment for multiple testing was applied. While multiple clinicopathological comparisons were performed, the primary aim was to identify potential associations for further investigation. Therefore, associations with *p*-values between 0.01 and 0.05 should be interpreted cautiously and warrant validation in independent cohorts. Since the fold change values representing the expression levels of miRNAs did not show a normal distribution, the correlation between these values was examined using the Spearman correlation test. The values of *p* < 0.05 were considered statistically significant.

## 3. Results

### 3.1. Patient Characteristics

The mean age of 86 CRC patients who participated in the present study was 58.425 years, with a standard deviation of ±14.79 years and a range from 30 to 79 years. Of the participants, 32 (37.2%) were female and 54 (62.8%) were male. Sixty-one tissue samples (70.9%) were obtained from the colon and 25 tissue samples (29.1%) were from the rectum. The tumor size was determined to be larger than 6 cm in 32 patients, which constitutes 37.2% of the study population. According to histopathologic tumor type, 69 (80.2%) cases were confirmed as adenocarcinoma and 17 (19.8%) as mucinous adenocarcinoma. While 14 (16.3%) patients had distant organ metastasis, 39 (45.3%) patients had lymph node metastasis (N1 + N2). In addition, neural invasion was observed in 17 (19.8%) patients and lymphovascular invasion in 24 (27.9%) patients. It was observed that 44 cases (51.2%) were classified as advanced stage disease (stage III–IV). The demographic and clinicopathological profiles of patients with CRC included in the study are summarized in Table 2.

### 3.2. miRNA Expression Levels

**Figure 1:**
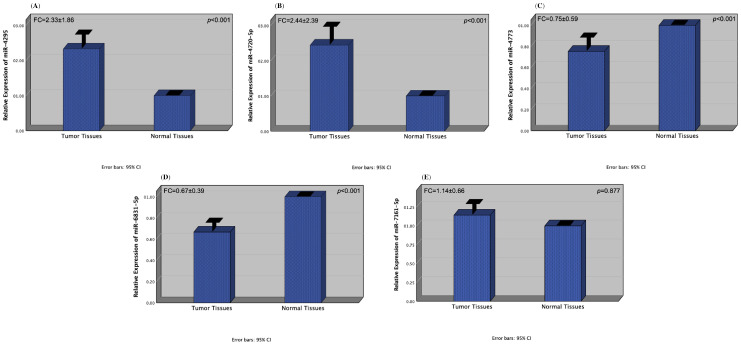
Expression patterns of miR-4295 (**A**), miR-4720-5p (**B**), miR-4773 (**C**), miR-6831-5p (**D**), and miR-7161-5p (**E**) in tumor and non-tumor tissues of CRC patients.

In our study, the expression levels of miR-4295, miR-4720-5p, miR-4773, miR-6831-5p, and miR-7161-5p were determined in a total of 172 tissue samples, including tumor and adjacent healthy tissues from 86 CRC patients. We found out that the expression levels of miR-4295 and miR-4720-5p were significantly increased in tumors compared to normal tissues (both *p*-values < 0.001) (fold-change values: 2.33 ± 1.86 and 2.44 ± 2.39, respectively) (Figure 1A,B). We also observed that the expression levels of miR-4773 and miR-6831-5p were significantly decreased in tumors compared to normal tissues (both *p* values < 0.001) (fold-change values: 0.75 ± 0.59 and 0.67 ± 0.39, respectively) (Figure 1C,D). Despite the increased expression levels of miR-7161-5p in pathological tissues compared to normal tissues, no significant difference was detected (*p* = 0.877) (fold-change value: 1.14 ± 0.66) (Figure 1E).

### 3.3. The Association of miRNA Expression with Clinical and Pathological Features in Patients with CRC

The relationship between the clinical and pathological characteristics of CRC patients and the expression levels of miR-4295, miR-4720-5p, miR-4773, miR-6831-5p, and miR-7161-5p was evaluated using Pearson’s Chi-squared (χ2) and Fisher’s exact tests, and the results of the analysis are shown in Table 3. As a result of these analyses, no significant correlation was found between the expression levels of miR-4295 and the clinical and pathological outcome of the patients. However, a significant correlation was found between the high expression of miR-4720-5p and the criteria of age (>55), tumor location (colon), and histological type (adenocarcinoma) (*p* values: 0.031, 0.049, and 0.018, respectively). However, there was a statistically significant lower expression of miR-4773 in patients with histological subtype adenocarcinoma compared to mucinous adenocarcinoma type (*p* = 0.025). In addition, a significant difference was observed between low expression of miR-6831-5p and tumor histological type of adenocarcinoma and tumor diameter of 6 cm or less (*p* values: 0.018 and 0.014, respectively). In accordance with the T1 + T2 categorization (T1: tumor has invaded the submucosa, T2: tumor has invaded the muscularis propria), the T3 + T4 categorization (T3: the tumor invades the peri-colorectal fatty tissue through the muscularis-propria, T4: tumor adhered to or invaded visceral peritoneum or adjacent organs or structures), and those with lymph node metastasis in the N0 (no regional lymph node metastasis) group showed statistical significance with high expression of miR-7161-5p compared to the N1 + N2 (N1: 1–3 pericolic lymph involvement, N2: 2–4 pericolic lymph involvement or perirectal involvement) group (*p* values; 0.025 and 0.018, respectively) (Table 3).

### 3.4. Correlation Between microRNAs Expression Levels

The results of the Spearman correlation test indicated a statistically significant positive correlation between the expression levels of miR-4295 and miR-4720-5p, and miR-4773 and miR-6831-5p (*p* < 0.001, r = 0.408; *p* < 0.001, r = 0.649, respectively), while a negative correlation was detected between miR-4720-5p and miR-6831-5p (*p* < 0.001, r = 0.245) (Table 4).

### 3.5. Gene Ontology (GO) Annotation and Kyoto Encyclopedia of Genes and Genomes (KEGG) Pathway Enrichment Analysis of miRNAs

To facilitate a comprehensive assessment of the biological pathways related to the candidate target genes of miR-4295, miR-4720-5p, miR-4773, and miR-6831-5p, GO functional annotation and KEGG pathway enrichment analyses were performed. The selection process involved the designation of three GO functional annotations: biological pathway (BP), cellular component, and molecular function (MF). GO analysis results presented that in terms of biological process, dysregulated miRNAs (miR-4295, miR-4720-5p, miR-4773, and miR-6831-5p) were primarily enriched in the cellular nitrogen compound metabolic process, biosynthetic process, cellular protein modification process, gene expression, cell–cell signaling, epidermal growth factor receptor signaling pathway, fibroblast growth factor receptor signaling pathway, etc. The cellular component was found to be enriched in organelles, protein complexes, the cytosol, and the nucleoplasm. The molecular functions that were primarily enriched were binding, protein binding, transcription factor activity, nucleic acid binding, transcription factor activity, enzyme binding, and enzyme regulator activity. As shown in Table 5, it was detected that 1201, 2068, and 1269 genes were linked to biological processes, cellular components, and molecular functions, respectively.

**Figure 2:**
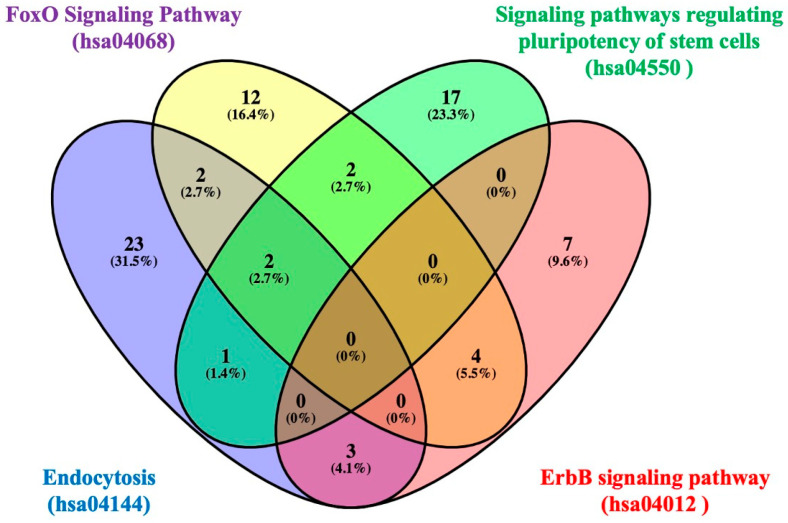
Venn diagram illustrating the common and pathway-specific target genes of four dysregulated miRNAs involved in CRC–associated pathways, including endocytosis, the FoxO signaling pathway, signaling pathways regulating pluripotency of stem cells, and the ErbB signaling pathway (the list of genes is reported in Supplementary Table S1).

**Figure 3:**
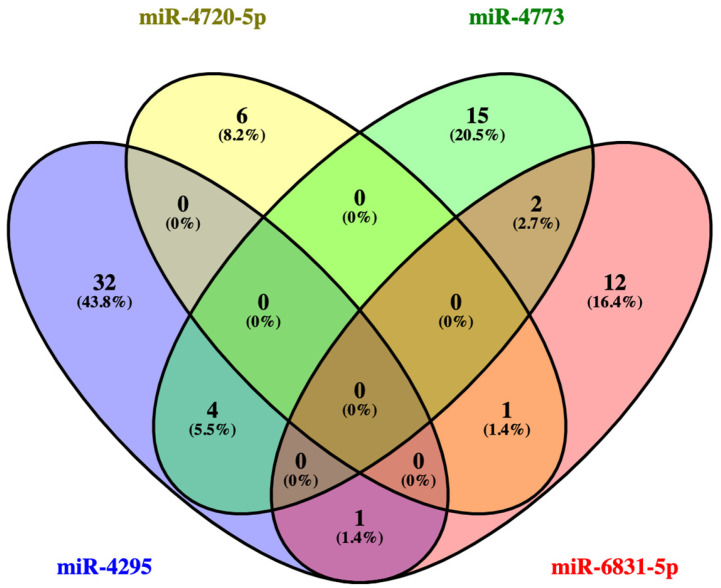
Venn diagram illustrating the intersection of target genes involved in common signaling pathways of dysregulated miRNAs (the list of genes is reported in Supplementary Table S2).

In order to investigate the functional mechanism of the dysregulated miRNAs, a KEGG pathway analysis was conducted using the DIANA-mirPath v.3 online tool. The results of this analysis indicated that the dysregulated miRNAs were significantly enriched in pathways involving endocytosis, the forkhead box O (FoxO) signaling pathway, signaling pathways regulating the pluripotency of stem cells, the ErbB signaling pathway, the TGF-beta signaling pathway, the mTOR signaling pathway, and so on. Table 6 summarizes the KEGG pathways that showed significant enrichment for the dysregulated miRNAs, together with the corresponding target genes linked to each pathway. A total of four dysregulated miRNAs were found to target six KEGG pathways, and it was observed that these target genes exhibited significant overlap between signaling pathways. The target genes of the four dysregulated miRNAs shown in Table 6 in four top-regulated pathways associated with CRC (endocytosis, FoxO signaling pathway, signaling pathways regulating pluripotency of stem cells, ErbB signaling pathway) were confirmed by Venn diagram. Bioinformatic analyses revealed that the common target genes of the three pathways (endocytosis, the FoxO signaling pathway, and the signaling pathways regulating the pluripotency of stem cells) are *SMAD2* and *IGF1R* (Figure 2 and Supplementary Table S1). To identify common regulatory mechanisms, signaling pathways involving the predicted targets of dysregulated miRNAs were examined, and Venn diagram analysis was used to display intersecting genes. In the signaling pathways given in Table 6, 37 target genes of miR-4295, 7 target genes of miR-4720-5p, 21 target genes of miR-4773, and 16 target genes of miR-6831-5p were detected. As demonstrated in Figure 3 and outlined in Supplementary Table S2, there was an overlap of four target genes (*CBLB*, *EPS15*, *SOD2*, *ABL2*) of “miR-4773” and “miR-4295”, one target gene (*WNT2B*) of “miR-4295” and “miR-6831-5p”, one target gene (*SMARCAD1*) of “miR-4720-5p” and “miR-6831-5p”, and two target genes (*TGFBR1, ACVR1C*) of “miR-4773” and “miR-6831-5p”.

## 4. Discussion

Colorectal carcinogenesis develops through the gradual accumulation of diverse genetic abnormalities accompanied by widespread epigenetic dysregulation in the otherwise normal colonic epithelium. As these alterations intensify over time, they facilitate the progression from initial mucosal changes to adenomatous formations and, ultimately, to invasive adenocarcinoma. While genomic mutations constitute a fundamental component in the initiation of this process, epigenetic modifications exert a substantial impact by shaping tumor behavior, influencing disease evolution, and contributing to malignant transformation [8,47]. Collectively, these features highlight colorectal cancer as a major public health issue, with mortality rates reaching approximately 9% in males and 8% in females [1]. Despite considerable advances in screening and therapeutic strategies, the long-term survival of patients with CRC remains unsatisfactory. The overall 5-year survival rate rarely exceeds 40%, and outcomes are substantially poorer in those diagnosed with metastatic disease, where 5-year survival drops to nearly 14%. In contrast, when CRC is detected at an early and localized stage, timely intervention can result in cure rates approaching 90%, underscoring the critical importance of effective early detection and intervention strategies [48,49]. Therefore, the identification and clinical implementation of novel molecular biomarkers are critically important for colorectal cancer, particularly given the inherent limitations of current screening and diagnostic modalities [50,51]. Accordingly, elucidating the key regulatory factors that drive CRC development and understanding the molecular mechanisms underlying disease progression are of paramount importance. Both genetic instability and epigenetic dysregulation contribute not only to the heterogeneous genotypic landscape of CRC but also act as fundamental drivers of tumor initiation and evolution. miRNAs, small non-coding RNAs expressed in a tissue- and time-specific manner, have emerged as critical post-transcriptional regulators in numerous pathophysiological processes. Recent evidence demonstrates that miRNAs participate in multiple stages of carcinogenesis, functioning either as tumor suppressors or as oncogenes depending on their biological context. Moreover, significant alterations in miRNA expression profiles have been widely reported across various malignancies and even in several non-cancerous diseases, highlighting their diagnostic and mechanistic relevance [52,53,54]. miRNAs are frequently located within fragile genomic regions and are highly vulnerable to genetic alterations, including point mutations, deletions, amplifications, and chromosomal translocations, all of which can disrupt their expression and contribute to tumorigenesis [55,56]. Numerous high-quality studies across diverse cancer types, including CRC, consistently demonstrate that miRNAs exhibit markedly altered expression patterns when comparing neoplastic tissues with their non-tumorous counterparts [57,58,59,60].

The present study was designed to investigate whether miR-4295, miR-4720-5p, miR-4773, miR-6831-5p, and miR-7161-5p contribute to colorectal carcinogenesis by analyzing their differential expression profiles in tumor and adjacent non-tumor tissues. miR-4295, which is located within the intronic region of the *VTI1A* gene on chromosome 10q25.2, is broadly expressed in vivo and represents a functionally versatile miRNA with diverse roles in cellular and molecular processes [61]. The current analysis demonstrated a significant elevation in miR-4295 expression in tumor tissues compared with adjacent non-tumorous tissues (*p* < 0.001). Several studies have also demonstrated that miR-4295 is upregulated in multiple cancer types and influences various tumor phenotypes. Consistent with our findings, elevated miR-4295 expression levels in tumor tissues compared to normal tissues have been reported in osteosarcoma [29] and glioma [30]. Yan et al. observed overexpression of miR-4295, which induces silencing of the *LRIG1* gene and overactivation of the epidermal growth factor receptor (EGFR)/phosphatidylinositol 3-kinase/Akt signaling pathway in gastric cancer, and therefore suggested it to have an oncogenic role [62]. Although Yang et al. reported a significant association between elevated miR-4295 expression and both tumor size and distant metastasis in gastric cancer tissues and cell lines, the present study did not demonstrate any significant correlation between miR-4295 overexpression in colorectal tumor tissues and the clinicopathological features of CRC patients. In the same study, the authors also showed that miR-4295 can directly bind to the 3′UTR of *PTEN*, markedly reducing its expression in vitro and thereby promoting cell proliferation, migration, and invasion in gastric cancer [32]. In another study, miR-4295 was shown to target *BTG1* in bladder cancer and suggested to be an oncogenic potential biomarker for the diagnosis and treatment of bladder cancer [33]. Liang et al. reported that the anti-tumor *PTPN14* gene was a potential target gene of miR-4295 in osteosarcoma and was negatively modulated by high miR-4295 levels [63]. Another study exhibited that miR-4295 expression was significantly increased in head and neck squamous cell carcinoma (HNSCC) tissues and cell lines compared to healthy tissues, promoting cell growth and metastasis, and was also associated with reduced patient survival [64]. On the other hand, a study of pancreatic ductal adenocarcinoma (PDAC) showed that miR-4295 expression was significantly increased and caused the suppression of its target gene *GPC5*. Through this feature, miR-4295 is likely to be utilized in developing treatments for PDAC [34]. According to the findings of our research and many other studies in other cancer types, the high expression of miR-4295 in neoplastic tissues, when evaluated together with its target genes, suggests that it exhibits an oncogenic function in CRC. Although the expression levels of miR-4295 have been demonstrated in many cancer types, the present research is the first to analyze its expression in CRC.

Our analysis revealed a statistically significant upregulation of miR-4720-5p in tumor tissues relative to normal tissues (*p* < 0.001), in agreement with findings reported in various previous studies. In one of the studies supporting our findings, Kim et al. reported that high expression of miR-4720-5p in patients with intramucosal gastric cancer (IMC) was strongly correlated with lymph node metastases. Consequently, the researchers proposed that it could serve as a valuable diagnostic marker for determining the metastatic predisposition of IMCs [36]. According to another study, among various miRNAs in hepatocellular carcinoma patients, only miR-4720-5p expression was found to be higher in blood serum than in whole blood [35]. In this study, we determined a significant correlation between high miR-4720-5p expression levels in tumor tissues and various clinicopathological data such as age (age > 55), anatomical location of the tumor (colon), and histopathological classification of the tumor (adenocarcinoma) (*p* < 0.05). The findings of the present study correspond to those of other research within the extant literature, which demonstrate that miR-4720-5p is significantly upregulated in tumor tissues. This finding indicates that miR-4720-5p may have a role as an oncogene in the pathogenesis of CRC.

In the current study, a downregulation of miR-4773 and miR-6831-5p was found in CRC (*p* < 0.001). In previous research, Sang et al. reported that high expression of *KIF4A*, *CENPF*, *OIP5*, *DEPDC1*, and *BUB1B* genes was linked to poor overall survival in breast cancer patients, and revealed that miR-4773 was involved in the negative regulation of these genes. In the same study, the researchers stated that low expression of miR-4773 provided new information about the causes of breast cancer metastasis and was likely to be a potential therapeutic target [38]. In a different study, researchers revealed that miR-4773 is one of the few downregulated miRNAs that directly inhibits the expression of *SMAD1* and *SMAD4*, the key transducers of osteogenic signaling in osteoblasts [65]. In contrast to the study performed by Tong et al. (2015), in which miR-4773 overexpression was observed in gastrointestinal stromal tumor (GIST) [37], we observed decreased expression of miR-4773 in malignant tissues in this study.

As the comprehensive literature reviewed within the present study shows, miR-6831-5p, the expression of which was detected below in our analysis, was also found by other researchers to have similar results in different cancer types. Kim et al. examined the relationship between IMC and miR-6831-5p and demonstrated that miR-6831-5p is markedly downregulated in malignant tissues compared with healthy counterparts. In the same study, specific genes in the *E2F* and AKT/mTOR signaling pathways were reported to be within the target genes of miR-6831-5p, while downregulation of miR-6831-5p was found to lead to an increase in the expression of these genes, thereby promoting carcinogenesis and metastasis [36]. A further study proved that pancreatic cancer was linked to reduced levels of miR-6831-5p, which was demonstrated to regulate the expression of the *TGFBR1* and *MAPK1* genes. These findings imply that low expression of miR-6831-5p may contribute to the upregulation of the *TGFBR1* and *MAPK1* genes, which may be associated with cancer development [40]. Contrary to the results of our study, Usuba et al. observed higher miR-6831-5p expression in the tumor tissue of bladder cancer patients compared to the control group of healthy individuals in a study with a different design [39]. We suppose that this difference may arise due to the use of a healthy control group. According to the results of our study, both miR-4773 and miR-6831-5p may function as tumor suppressors in CRC due to decreased expression levels in tumor tissues.

Although the expression level of miR-7161-5p did not show a significant difference between tumors and adjacent non-tumor tissues, the inclusion of this finding in the study is noteworthy, as such findings contribute to improving the specificity of miRNAs involved in colorectal carcinogenesis. Furthermore, its association with clinical-pathological parameters such as lymph node metastasis and invasion status suggests that it has the potential to require further investigation.

A single miRNA molecule can target hundreds of different types of mRNA and can affect the expression of many genes that often functionally interact with these mRNAs. The identification of miRNA-regulated signaling pathways and cellular processes involved in colorectal carcinogenesis using bioinformatics tools will provide important insights and improve our understanding of the complex and multi-stage molecular pathogenesis of CRC. In the present study, a KEGG pathway analysis of dysregulated miRNAs was conducted, which revealed that major signaling pathways, including endocytosis, FoxO, ErbB, TGF-beta, and mTOR, might be affected by dysregulated miRNAs. The endocytosis pathway is known to play a role in the development, progression, and regulation of cancer, and the proteins that play important roles in this signaling pathway have been associated with CRC [66,67]. FoxO transcription factors constitute a regulatory protein family involved in the control of diverse cellular processes such as cell growth, differentiation, programmed cell death, and responses to oxidative stress. FoxO transcription factors are among the key molecules that regulate a variety of biological processes during the development of CRC [68]. Furthermore, the ErbB receptor tyrosine kinase family, the TGF-beta signaling pathway, and the mTOR signaling pathway, also known as the mammalian target of rapamycin, have also been identified as important factors in the development and progression of CRC [69,70,71].

Differential expression analysis alone provides valuable insights into the potential role of miRNAs in colorectal carcinogenesis, but it does not directly indicate the functional effects of these miRNAs. To overcome this limitation, bioinformatic approaches were employed in this study using the DIANA-miRPath online database to perform target prediction and pathway enrichment analyses. This revealed that the differentially expressed miRNAs are associated with several important cancer-related signaling pathways, including the TGF-β, mTOR, ErbB, FoxO, and endocytosis pathways. These findings revealed possible molecular mechanisms that could explain the effects of these miRNAs in CRC.

Clinically, the findings of this study suggest that irregularly expressed miRNAs may have potential value as molecular biomarkers in CRC. The significant upregulation of miR-4295 and miR-4720-5p and the downregulation of miR-4773 and miR-6831-5p in tumor tissues suggest that these miRNAs play important roles in the formation of colorectal carcinogenesis and could be considered as candidate diagnostic/prognostic biomarkers. Furthermore, the relationship of specific miRNAs with clinical/pathological data such as tumor location, histological subtype, and tumor size further highlights their potential clinical importance. Along with the necessity for additional studies such as functional validation, these results provide a basis for future studies investigating the importance of these miRNAs in the development of early diagnosis, risk classification, and miRNA-based treatment strategies in CRC. This study has several limitations. Firstly, no experimental analysis was performed to identify the genes targeted by miRNAs. Therefore, further studies can be performed to identify the target genes of miR-4295, miR-4720-5p, miR-4773, and miR-6831-5p that are effective in CRC. Additionally, the patient cohort was obtained from a single center, which may limit the generalizability of the findings to larger populations. The sample size of the study may not be sufficient to reveal the association between the clinicopathological data of the patients and the expression levels of miRNAs. Furthermore, certain clinical-pathological subgroups, such as patients consuming alcohol, include a relatively smaller number of subjects, which may reduce statistical power and increase the risk of type II error. Therefore, the observed relationships should be interpreted with caution and confirmed in larger, multicenter studies. Moreover, the effects of miRNAs on cell proliferation, invasion, metastasis, and apoptosis, inducing CRC development, have not yet been investigated in vitro. Additional validation studies, including gain-of-function and loss-of-function assays using functional in vitro experiments, will be valuable in confirming the regulatory roles of these miRNAs and their direct target genes in CRC. Although the present research has several limitations, the results will contribute to future studies to reveal the genetic mechanisms involved in determining the relationship between miRNAs and colorectal carcinogenesis.

## 5. Conclusions

In conclusion, the findings of this study suggest that miR-4295 and miR-4720-5p may be associated with oncogenic processes, while miR-4773 and miR-6831-5p may be linked to tumor-suppressing mechanisms in CRC. These interpretations are primarily based on differential expression patterns and bioinformatics analyses. Although these miRNAs showed significant dysregulation in tumor tissues, functional studies are required to confirm their precise biological roles. Furthermore, to elucidate the molecular and cellular pathophysiology of CRC and develop new diagnostic and therapeutic models, more research is needed with larger patient cohorts in different populations. It is hoped that the creation of bioinformatics-supported miRNA-regulated networks will facilitate the development of new treatment methods for CRC and highlight the potential value of these miRNAs as candidate molecular biomarkers.

## Figures and Tables

**Table 1:** Primer sequences of miRNA.

miRNA	Primer	Sequence (5′–3′).
miR-4295	Forward	GCAGCAGTGCAATGTTTTC
	Reverse	GGTCCAGTTTTTTTTTTTTTTTAAGG
miR-4720-5p	Forward	CAGCCTGGCATATTTGGT
	Reverse	AGGTCCAGTTTTTTTTTTTTTTTAAGT
miR-4773	Forward	CAGCAGAACAGGAGCATAG
	Reverse	TCCAGTTTTTTTTTTTTTTTGCCTT
miR-6831-5p	Forward	GGTAGAGTGTGAGGAGGAG
	Reverse	GGTCCAGTTTTTTTTTTTTTTTGAC
miR-7161-5p	Forward	GCAGTAAAGACTGTAGAGGCA
	Reverse	TCCAGTTTTTTTTTTTTTTTACCAGTT
*RNU6*	Forward	GCTTCGGCAGCACATATACTAAAAT
	Reverse	CGCTTCACGAATTTGCGTGTCAT

**Table 2:** Baseline demographic and clinicopathological characteristics of the CRC cohort.

Characteristics	Patients N (%)
**Age (years)**	
≥55	53 (61.6)
<55	33 (38.4)
**Gender**	
Male	54 (62.8)
Female	32 (37.2)
**Cigarette smoking**	
Yes	26 (30.2)
No	60 (69.8)
**Alcohol drinking**	
Yes	4 (4.7)
No	82 (95.3)
**Tumor location**	
Colon	61 (70.9)
Rectum	25 (29.1)
**Invasion**	
T1 + T2	16 (18.6)
T3 + T4	70 (81.4)
**Neural invasion**	
Yes	17 (19.8)
No	69 (80.2)
**Lymphovascular invasion**	
Yes	24 (27.9)
No	62 (72.1)
**Distant metastasis**	
M0	71 (83.7)
M1	14 (16.3)
**Lymph node metastasis**	
N0	47 (54.7)
N1 + N2	39 (45.3)
**Tumor stage**	
I–II	42 (48.8)
III–IV	44 (51.2)
**Tumor size (cm)**	
≤6	54 (62.8)
>6	32 (37.2)
**Tumor histological type**	
Adenocarcinoma	69 (80.2)
Mucinous adenocarcinoma	17 (19.8)

(N0, no regional lymph node metastasis; N1, 1–3 pericolic lymph node involvement; N2, 2–4 pericolic lymph node involvement or perirectal involvement; M0, no distant metastasis; M1, metastasis in more than one organ/region or peritoneum; T1, tumor invading the submucosa; T2, tumor invading the muscularis propria; T3, tumor invading the pericolorectal fatty tissue by crossing the muscularis propria; T4, tumor adhering to or invading the visceral peritoneum or adjacent organs or structures).

**Table 3:** Relationship between clinicopathological parameters of CRC patients and miRNA expression profiles.

Variables	miR-4295			miR-4720-5p			miR-4773			miR-6831-5p			miR-7161-5p		
	Low	High	*p*	Low	High	*p*	Low	High	*p*	Low	High	*p*	Low	High	*p*
**Age (years)**															
≥55	10	43	0.427	16	37	0.031 *	41	12	0.797	46	7	0.550	28	25	1.000
˂55	9	24		3	30		24	9		27	6		17	16	
**Gender**															
Male	14	40	0.297	10	44	0.420	44	10	0.122	49	5	0.064	31	23	0.267
Female	5	27		9	23		21	11		24	8		14	18	
**Cigarette smoking**															
Yes	9	17	0.090	5	21	0.782	19	7	0.787	24	2	0.327	13	13	0.817
No	10	50		14	46		46	14		49	11		32	28	
**Alcohol drinking**															
Yes	1	3	1.000	0	4	0.571	4	0	0.568	4	0	1.000	1	3	0.344
No	18	64		19	63		61	21		69	31		44	38	
**Tumor location**															
Colon	15	46	0.568	17	44	0.049 *	45	16	0.594	50	11	0.330	33	28	0.641
Rectum	4	21		2	23		20	5		23	2		12	13	
**Invasion**															
T1 + T2	2	14	0.505	5	11	0.332	9	7	0.058	14	2	1.000	4	12	0.025 *
T3 + T4	17	53		14	56		56	14		59	11		41	29	
**Neural invasion**															
Yes	2	15	0.340	3	14	0.753	12	5	0.753	14	3	0.715	9	8	1.000
No	17	52		16	53		53	16		59	10		36	33	
**Lymphovascular invasion**															
Yes	5	19	1.000	5	19	1.000	19	5	0.782	19	5	0.502	11	13	0.480
No	14	48		14	48		46	16		54	8		34	28	
**Distant metastasis**															
M0	17	55	0.726	15	57	0.500	55	17	0.738	62	10	0.437	39	6	0.562
M1	2	12		4	10		10	4		11	3		33	8	
**Lenf node metastasis**															
N0	10	37	1.000	10	37	1.000	32	15	0.085	39	8	0.764	19	28	0.018 *
N1 + N2	9	30		9	30		33	6		34	5		26	13	
**Tumor stage**															
I-II	10	32	0.797	9	33	1.000	29	13	0.212	35	7	0.769	18	24	0.130
III-IV	9	35		10	34		36	8		38	6		27	17	
**Tumor size (cm)**															
≤6	14	40	0.297	12	42	1.000	40	14	0.797	50	4	0.014 *	27	27	0.657
>6	5	27		7	25		25	7		23	9		18	14	
**Tumor histological type**															
Adenocarcinoma	15	54	1.000	19	50	0.018 *	56	13	0.025 *	62	7	0.018 *	39	30	0.175
Mucinous adenocarcinoma	4	13		0	17		9	8		11	6		6	11	

*: *p* value is significant at the 0.05 level (2-tailed).

**Table 4:** Correlation between miRNAs.

		miR-4295	miR4720-5p	miR-4773	miR-6831-5p
miR-4295	r	1.000	0.408 **	0.001	−0.140
	*p* value	.	<0.001	0.993	0.067
miR-4720-5p	r	0.408 **	1.000	−0.143	−0.245 **
	*p* value	<0.001	.	0.062	<0.001
miR-4773	r	0.001	−0.143	1.000	0.649 **
	*p* value	0.993	0.062	.	<0.001
miR-6831-5p	r	−0.140	−0.245 **	0.649 **	1.000
	*p* value	0.067	<0.001	<0.001	.

r: Correlation Coefficient; **: Correlation is significant at the 0.01 level (2-tailed).

**Table 5:** Gene Ontology (GO) enrichment analysis of target genes associated with miR-4295, miR-4720-5p, miR-4773, and miR-6831-5p across biological process, cellular component, and molecular function categories.

**Biological Process**			
**GO ID**	**GO term name**	***p*** **-value**	**Target gene counts**
GO:0034641	Cellular nitrogen compound metabolic process	3.47817584956 × 10^−30^	425
GO:0009058	Biosynthetic process	3.93511188734 × 10^−22^	360
GO:0006464	Cellular protein modification process	3.76953500217 × 10^−14^	214
GO:0010467	Gene expression	5.78640712256 × 10^−11^	64
GO:0007267	Cell-cell signaling	3.30789780557 × 10^−9^	78
GO:0038095	Fc-epsilon receptor signaling pathway	1.47657403515 × 10^−8^	25
GO:0007268	Synaptic transmission	4.71710702933 × 10^−7^	51
GO:0048011	Neurotrophins TRK receptor signaling pathway	1.12711148506 × 10^−6^	30
GO:0065003	Macromolecular complex assembly	1.55152673455 × 10^−6^	85
GO:0022607	Cellular component assembly	5.97672344625 × 10^−6^	114
GO:0007173	Epidermal growth factor receptor signaling pathway	1.76728381298 × 10^−5^	28
GO:0008543	Fibroblast growth factor receptor signaling pathway	0.000386817261063	25
GO:0006461	Protein complex assembly	0.000386817261063	70
GO:0048015	Phosphatidylinositol-mediated signaling	0.000434426966324	20
GO:0043687	Post-translational protein modification	0.0010738403889	19
GO:0006351	Transcription, DNA-templated	0.00142425137299	206
GO:0006950	Response to stress	0.00149198607409	172
GO:0009056	Catabolic process	0.00163676996544	144
GO:0006367	Transcription initiation from RNA polymerase II promoter	0.00299682613464	26
GO:0007179	Transforming growth factor beta receptor signaling pathway	0.0038006703892	25
GO:0008219	Cell death	0.00476511399854	76
GO:0000278	Mitotic cell cycle	0.00585721465851	33
GO:0044267	Cellular protein metabolic process	0.0111545812275	36
GO:0016032	Viral process	0.0116813266127	36
GO:0061024	Membrane organization	0.0121627885727	48
GO:0044281	Small molecule metabolic process	0.0121710803496	160
GO:0009887	Organ morphogenesis	0.0129795649994	24
GO:0044403	Symbiosis, encompassing mutualism through parasitism	0.0218844710588	39
GO:0016070	RNA metabolic process	0.0392326950386	22
GO:0000288	Nuclear-transcribed mRNA catabolic process, deadenylation-dependent decay	0.0484397081143	9
GO:0008150	Biological process	0.0290099521007	1201
**Cellular Component**			
**GO ID**	**GO term name**	***p*** **-value**	**Target gene counts**
GO:0043226	Organelle	1.00780607172 × 10^−48^	820
GO:0043234	Protein complex	1.24440475458 × 10^−10^	322
GO:0005829	Cytosol	1.00963526144 × 10^−5^	222
GO:0005654	Nucleoplasm	2.52204325661 × 10^−5^	104
GO:0005575	Cellular component	1.0841859996 × 10^−6^	1268
**Molecular Function**			
**GO ID**	**GO term name**	***p*** **-value**	**Target gene counts**
GO:0043167	Ion binding	1.98593181043 × 10^−21^	492
GO:0000988	Protein binding transcription factor activity	2.80440576603 × 10^−11^	64
GO:0001071	Nucleic acid binding transcription factor activity	1.02377765451 × 10^−5^	90
GO:0019899	Enzyme binding	1.02377765451 × 10^−5^	113
GO:0030234	Enzyme regulator activity	0.029056114833	68
GO:0003674	Molecular function	8.97234178148 × 10^−10^	1269

**Table 6:** KEGG analysis of dysregulated miRNAs in tissues of CRC patients.

Pathways	Pathways ID	miRNAs	Target Genes	*p*-Value
Glioma	hsa05214	miR-4295	*SOS2*, *TGFA*, *CALM2*, *SOS1*, *IGF1*, *CDKN1A*, *PTEN*	0.0013
		miR-4720-5p	*CAMK2A*	
		miR-4773	*CAMK2D*, *CDK6, E2F3*	
		miR-6831-5p	*BRAF, IGF1R*	
Endocytosis	hsa04144	miR-4295	*RNF41*, *ARAP2*, *MET*, *SMURF2*, *CLTC*, *CHMP4B*, *ASAP1*, *RAB5A*, *CBLB*, *EPS15*, *ZFYVE9*, *HSPA8*, *LDLR*, *TGFBR2*	0.0017
		miR-4720-5p	*AGAP1*, *ERBB3*	
		miR-4773	*TGFBR1*, *SMAD2*, *CHMP4C*, *CAV2*, *CBLB*, *TRAF6*, *EPS15*, *DNM1*, *PIP5K1A*, *STAMBP*, *FGFR2*, *KDR*, *PARD6B*, *ERBB4*	
		miR-6831-5p	*TGFBR1*, *SMAD6*, *IGF1R*, *ADRB3*	
FoxO signaling pathway	hsa04068	miR-4295	*RAG1*, *SOS2*, *PRKAA2*, *GADD45A*, *S1PR1*, *SOS1*, *PRKAA1*, *IGF1*, *SOD2*, *CDKN1A*, *PTEN*, *CCNG2*, *TGFBR2*, *BCL2L11*	0.0114
		miR-4720-5p	*PRKAG1*	
		miR-4773	*TGFBR1*, *SMAD2*, *SMAD4*, *SOD2*	
		miR-6831-5p	*BRAF*, *TGFBR1*, *IGF1R*, *NLK*, *PDPK1*	
Signaling pathways regulating pluripotency of stem cells	hsa04550	miR-4295	*JARID2*, *INHBB*, *HOXB1*, *WNT2B*, *INHBA*, *ACVR1*, *SMAD5*, *IGF1*	0.0137
		miR-4720-5p	*SMARCAD1*, *JAK2*	
		miR-4773	*SMAD2*, *PCGF5*, *SMAD4*, *LIFR*, *ACVR1C*, *FGFR2*	
		miR-6831-5p	*HAND1*, *SMARCAD1*, *WNT2B*, *IGF1R*, *FZD3*, *ACVR2B*, *SKIL*, *ACVR1C*, *WNT9A*	
Glycosylphosphatidylinositol (GPI)-anchorbio synthesis	hsa00563	miR-4295	*PGAP1*, *PIGP*, *PIGA*	0.0190
		miR-4720-5p	*PIGB*, *PIGA*	
		miR-4773	*PYURF*	
		miR-6831-5p	*PIGN*	
ErbB signaling pathway	hsa04012	miR-4295	*SOS2*, *TGFA*, *CBLB*, *SOS1*, *CDKN1A*, *ABL2*, *EREG*	0.0244
		miR-4720-5p	*ERBB3*, *CAMK2A*, *NRG1*	
		miR-4773	*CAMK2D*, *CBLB*, *ABL2*, *ERBB4*	
		miR-6831-5p	*BRAF*, *RPS6KB2*	
TGF-beta signaling pathway	hsa04350	miR-4295	*INHBB*, *SMURF2*, *INHBA*, *ACVR1*, *SKP1*, *ZFYVE9*, *SMAD5*, *TGFBR2*	6.48 × 10^−9^
		miR-4773	*TGFBR1*, *SMAD2*, *SMAD4*, *TFDP1*, *ACVR1C*, *PPP2R1B*	
		miR-6831-5p	*TGFBR1*, *SMAD6*, *RPS6KB2*, *ACVR2B*, *E2F5*, *GDF6*, *ACVR1C*	
Morphine addiction	hsa05032	miR-4295	*GABRA1*, *ADCY1*, *GABRA3*, *KCNJ6*	1.99 × 10^−6^
		miR-4773	*PDE1C*, *GABBR2*, *GABRA5*, *GABRP*	
		miR-6831-5p	*GNB3*, *ADORA1*, *KCNJ6*, *GABRR1*, *GNG5*	
GABAergic synapse	hsa04727	miR-4295	*GABRA1*, *ADCY1*, *GABRA3*, *KCNJ6*	4.93 × 10^−6^
		miR-4773	*SLC38A1*, *GPHN*, *GABBR2*, *GABRA5*, *GAD2*, *GABRP*, *SLC6A13*, *SLC6A1*	
		miR-6831-5p	*GNB3*, *TRAK2*, *GLUL*, *KCNJ6*, *GABRR1*, *GNG5*	
Mtor signaling pathway	hsa04150	miR-4295	*TSC1*, *RRAGD*, *PRKAA2*, *PRKAA1*, *IGF1*, *EIF4E2*, *PTEN*, *ULK2*	0.0244
		miR-4773	*TSC1*	
		miR-6831-5p	*BRAF*, *RPS6KB2*, *RICTOR*, *ULK3*, *PDPK1*	

## Data Availability

The datasets used and/or analyzed during the current study are available from the corresponding author on reasonable request.

## Supplementary Materials

The following supporting information can be downloaded at: https://www.mdpi.com/article/10.3390/diagnostics16040614/s1, Table S1: Common target genes of signal pathways; Table S2: Common target genes of miRNAs.

## Abbreviations

The following abbreviations are used in this manuscript:

APC	Adenomatous polyposis coli
Ct	Threshold cycle
CRC	Colorectal cancer
ErbB	ErbB receptor
EGFR/MAPK	Epidermal growth factor receptor/mitogen-activated protein kinase
FoxO	Forkhead box O
GO	Gene Ontology
HNSCC	Head and neck squamous cell carcinoma
Hippo-YAP	Hippo signaling pathway with yes-associated protein
IMC	Intramucosal gastric cancer
JAK/STAT	Janus kinase/signal Transducer and activator of transcription
KEGG	Kyoto Encyclopedia of Genes and Genomes
miRNA	microRNA
mTOR	Mechanistic (mammalian) target of rapamycin
PDAC	Pancreatic ductal adenocarcinoma
PI3K/AKT	Phosphoinositide 3-kinase/protein kinase B (AKT)
RT-qPCR	Quantitative real time PCR
TGF-beta	Transforming growth factor beta
VEGF/VEGFR	Vascular endothelial growth factor/vascular endothelial growth factor receptor
3′UTR	3′ untranslated region
